# Prevalence and risk factors of *Ascaris lumbricoides* (Linnaeus, 1758)*, Trichuris trichiura* (Linnaeus, 1771) and HBV infections in Southwestern China: a community-based cross sectional study

**DOI:** 10.1186/s13071-015-1279-2

**Published:** 2015-12-24

**Authors:** Peng-Lei Xiao, Yi-Biao Zhou, Yue Chen, Ya Yang, Yan Shi, Jian-Chuan Gao, Wu-Li Yihuo, Xiu-Xia Song, Qing-Wu Jiang

**Affiliations:** Fudan University School of Public Health, Building 8, 130 Dong An Road, Xuhui District, Shanghai, 200032 China; Key Laboratory of Public Health Safety, Fudan University, Ministry of Education, Building 8, 130 Dong An Road, Xuhui District, Shanghai, 200032 China; Fudan University Center for Tropical Disease Research, Building 8, 130 Dong An Road, Xuhui District, Shanghai, 200032 China; School of Epidemiology, Public Health and Preventive Medicine, Faculty of Medicine, University of Ottawa, 451 Smyth Road, Ottawa, Ontario K1H 8M5 Canada; Puge Center for Disease Prevention and Control, 4 Qing Nian Road, Puge County, Liangshan Prefecture, Sichuan Province, 615399 China; Department of Epidemiology, School of Public Health, Fudan University, Room 307, Building 8, 130 Dong An Road, Xuhui District, Shanghai, 200032 China

**Keywords:** HBV, *Ascaris lumbricoides*, *Trichuris trichiura*, Faeces, Co-infection

## Abstract

**Background:**

Intestinal helminths do not cause severe diseases in general, however, when combined with other diseases such as immunodeficiency diseases, there would be massive complications. Infections with Hepatitis B Virus (HBV) may lead to immunological disturbances and defects of cellular immunity and there is a need of clarification whether HBV infections are associated with infections with intestinal helminths.

**Methods:**

A community-based cross sectional study was conducted in Tezi town, Puge County of the Liangshan Prefecture, southwestern China from October 23rd to November 3rd, 2014. Four hundred and thirty eight people (median age = 37 years, IQR = 22–49) were enrolled in this study. Modified Kato-Katz thick smear was used to detect intestinal helminths. HBV DNA was quantified to confirm HBV infection.

**Results:**

Among the 438 participants, 9.1 %, 13.5 % and 30.6 % were infected with HBV, *A. lumbricoides* (L., 1758) and *T. trichiura* (L., 1771)*,* respectively; 7.1 % (30/438) were infected with both *A. lumbricoides* and *T. trichiura* and 2.3 % (10/438) were co-infected with HBV and *A. lumbricoides*. The multivariate logistic regression analysis showed that age (21–30 years versus >50 years: OR = 6.66, 95 % CI = 2.15–20.68), drug abuse (OR = 6.96, 95 % CI = 1.11–43.90), *A. lumbricoides* infection (OR = 3.60, 95 % CI = 1.48–8.75), fertilization with faeces after disposal (OR = 0.15, 95 % CI = 0.04–0.47) and working on a farm (OR = 4.59, 95 % CI = 1.44–14.63) were significantly associated with HBV infection. Having toilets at home was negatively related to *A. lumbricoides* infection (OR = 0.52, 95 % CI = 0.27–0.98) and *T. trichiura* infection (OR = 0.48, 95 % CI = 0.28–0.80).

**Conclusions:**

*Ascaris lumbricoides* was independently associated with HBV infection, and faeces might be the medium of HBV transmission. Improving hygiene conditions and habits are essential to reduce the risks of *A. lumbricoides* and *T. trichiura* infections.

## Background

Intestinal helminths are common in developing countries including China especially in rural areas due to the poor economy and sanitation conditions. Globally, about two billion people are infected with at least one of the soil-transmitted helminth (STH) species, particularly in developing countries with poor socioeconomic status [1]. Intestinal helminths do not cause severe diseases in general; however, when combined with other diseases like immunodeficiency diseases, there could be massive complications causing some serious consequences [2]. Some studies have reported that human immunodeficiency virus (HIV) increases the risk of helminthic infection [3] as HIV attacks the human immune system and causes cellular immunity dysfunction. For example, people infected with HIV were more likely to be infected with parasites, like *Cryptosporidium*, compared with those who were HIV negative [4]. Similarly, HBV infection could lead to immunological disturbances and defects of cellular immunity [5]. However, it needs to be clarified whether HBV infection is associated with helminth infections.

*Ascaris lumbricoides* and *T. trichiura* occur at high prevalence in rural areas of China [6]. At the same time, HBV infection is not uncommon in such places [7]. STH infections cause a loss of 39 million disability-adjusted life-years (DALYs), comparable to malaria or tuberculosis [8, 9]. It is estimated that two billion people have either past or present infection with HBV in the world, and 240 million are chronic carriers of HBV surface antigen (HBsAg) [7]. In China, HBsAg carry rate was estimated to be 7.18 % for people under 60 years of age in 2006 [10]. It has been indicated that HBV infection was associated with *Schistosoma japonicum*. The immunomodulation due to schistosome infections might restrict immune control of HBV leading to more severe viral infections [11]. So far few studies have investigated the relationship between HBV infection and intestinal helminths. In this study, we aimed to determine the prevalence and risk factors of infections with intestinal helminths including *A. lumbricoides* and *T. trichiura* and HBV infection in a rural community of southwestern China. We also tried to explore the association between HBV infection and *A. lumbricoides* and *T. trichiura*.

## Methods

### Study field

**Fig. 1:**
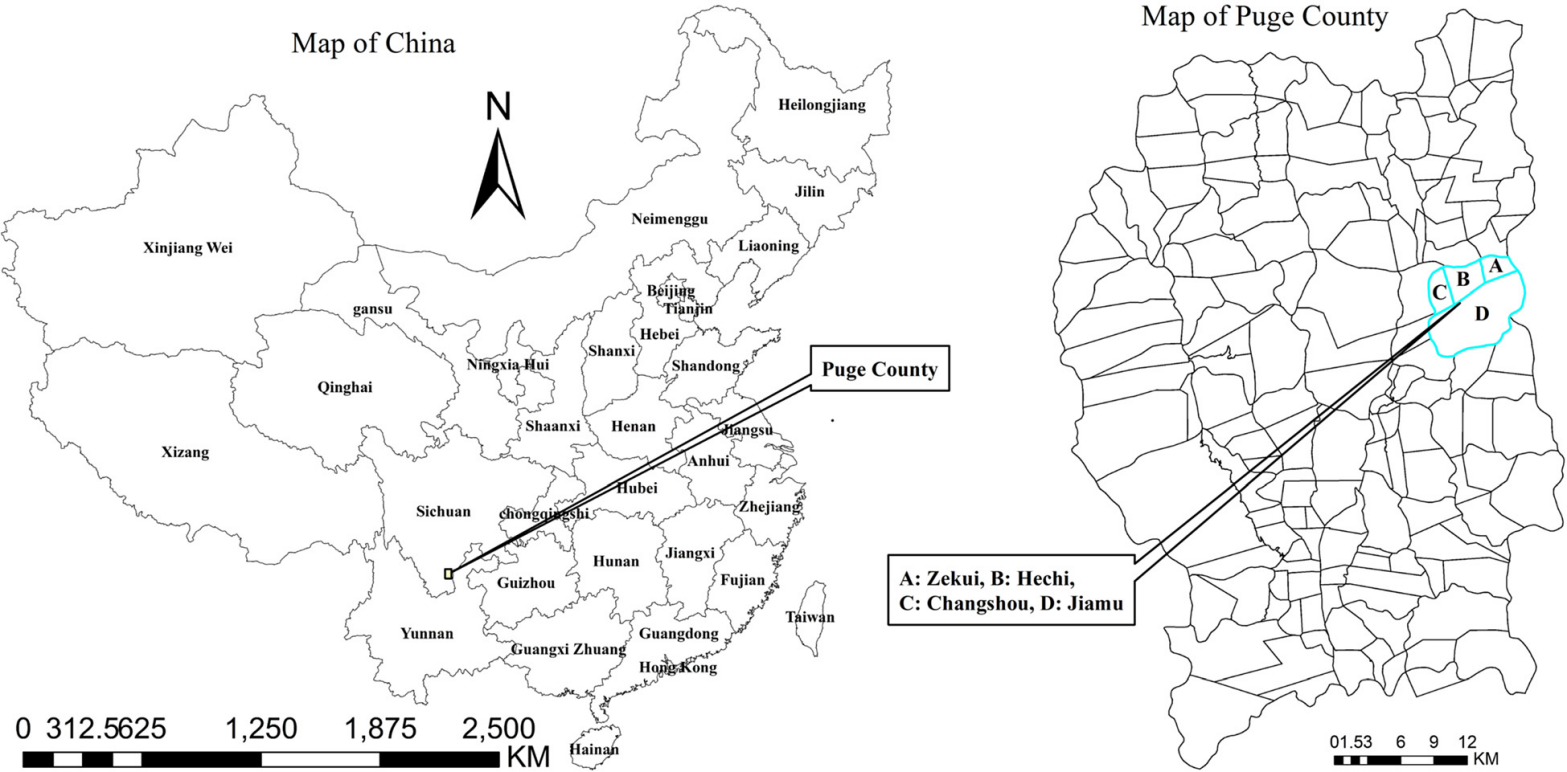
Map of the study area, showing the Puge County and four villages. Villages 1–4 are labelled as A, B, C and D (Zekui, Hechi, Changshou and Jiamu, respectively) in the map of the Puge County

A community-based cross-sectional study was conducted in Tezi town (27°36′39.43″N, 102°40′38.31″E; altitude: 2,147 m), Puge County of the Liangshan Prefecture (Fig. 1), southwestern China from October 23rd to November 3rd, 2014. This town covers an area of 50.2 km^2^, with a population of approximately 4,000 inhabitants. Tezi is an underdeveloped region inhabited by the Yi people, an ethnic minority group in China. Lavatories, latrines or any other form of sanitation facilities were not available before 2006. Both human and domestic animal faeces were left untreated [12]. The current study included people aged 6 years or more, living in the area for more than 6 months every year. The participants had no severe organic or mental diseases and pregnant women were excluded. A total of 645 individuals were randomly selected from the four villages (Zekui village, Hechi village, Jiamu village and Changshou village which were numbered as village 1–4 (see the map of Puge County in Fig. 1).

### Study procedures

All the health workers participating in this investigation were staff of the local Center for Disease Control and Prevention (CDC) and were trained under the guidance of a unified protocol. Then health workers informed all the potential participants according to the protocol list and carefully explained the objectives, procedures and potential risks of the study.

All the residents sampled were screened for HBV surface antigen by using the Diagnostic Test Kit for HBV (colloidal gold) (product of Livzon Pharmaceutical Group Inc., Zhuhai, P. R. China, batch number: 2014080200, 100 persons per kit). Those with positive HBV surface antigen were asked to provide 5 ml blood specimen for HBV DNA testing by using the diagnostic kit for quantification of HBV DNA (PCR-Fluorescence Probing) (product of Daan Gene Group Inc., Zhongshan, P. R. China, batch number: 2015002, 20 persons per kit).

The participants were given one faeces collection container and were asked to provide their faecal samples of at least 30 g collected in the morning at home. An oral description and specific instructions for handling and contamination avoidance of the stool samples were given to all the participants who gave consent. A questionnaire was administered and covered the information on socio-demographic factors, concomitant physical health, hygienic habits, farm work, drug abuse, and high-risk sex behaviours. All the samples were sent to the laboratory of the local CDC for examination as soon as possible after they were collected. The faecal samples were processed within 24 h post-collection by using the modified Kato-Katz thick smear (a semi-quantitative faeces examination technique) for detection of helminth eggs. Three smears of each faecal sample were prepared for examination. Every smear was initially read by two examiners and reviewed by a third examiner if there was disagreement.

### Statistical analysis

Data were double-entered and crosschecked by using the EpiData software (version 3.1; The EpiData Association, Odense, Denmark). Descriptive summary measures of frequency and central tendency of participants’ characteristics were computed. In the univariate analyses, the Pearson χ^2^ tests were used to examine the associations of participants’ characteristics with HBV, *A. lumbricoides* or *T. trichiura* by computing crude odds ratios (ORs) with 95 % confidence intervals (CIs). Logistic regression model was subsequently employed for multivariate analysis and adjusted ORs with 95 % CIs were calculated for the risk factors identified. A two-sided P value of 0.05 or less was regarded as significant. Statistical analyses were carried out with the SPSS statistical package (version 17.0; IBM SPSS Institute, Inc., USA).

### Ethical statement

This study was evaluated and approved by the Ethics Review Committee of the Ethical Institute of The School of Public Health, Fudan University. The potential participants who agreed to attend the study were asked to sign a written informed consent by the staff of the local CDC. If participants were less than 18 years of age, their parents were asked to sign a written parental permission. At the completion of the study and in accordance with the local treatment policies anti-helminthic treatment was offered for free to all participants found to be infected with intestinal helminths through the local CDC*.*

## Results

There were 645 participants in the study. After the exclusion of participants whose faeces or blood samples were not collected or who did not complete the questionnaires, 438 participants were finally included in the analysis. Those excluded (median age = 32 years, interquartile range (IQR) = 14–44) were significantly younger (*P* = 0.003) than those included in the analysis (median age = 37 years, IQR = 22–49). There were no significant differences between them in gender, occupation, race, education and household income. Table 1 shows the characteristics of the participants. Among 438 participants, 39.3 % were male, 96.8 % were of Yi nationality, 65.3 % were illiterate and 22.8 % were unmarried. Infection rates of HBV, *A. lumbricoides* and *T. trichiura* were 9.1, 13.5 and 30.6 % respectively. Of these, 7.1 % (30/438) had a co-infection of *A. lumbricoides* and *T. trichiura*, 2.3 % (10/438) had a co-infection of HBV and *A. lumbricoides,* and 2.7 % (12/438) had a co-infection of HBV and *T. trichiura* (Table 2).

**Table 1:** Characteristics of the study population

Variables/ states		No. individuals	Proportion in %
Age (years)	6–10	54	12.3
	11–20	48	11.0
	21–30	66	15.1
	31–40	86	19.6
	41–50	88	20.1
	>50	96	21.9
Gender	Male	172	39.3
	Female	266	60.7
Occupation	Worker	4	0.9
	Peasant	370	84.5
	Else	64	14.6
Education	Illiterate	286	65.3
	Elementary school and above	152	34.7
Marital status	Unmarried	100	22.8
	First married	293	66.9
	Divorced and other	45	10.3
Home income (¥yuan, RMB)	<1,000	12	2.7
	1,000–2,999	71	16.2
	3,000–4,999	142	32.4
	5,000–9,999	75	17.1
	≥10,000	138	31.
Drug abuse	Yes	9	2.1
	No	429	97.9
Work on farm	Yes	265	60.5
	No	113	29.5
Clean food when eaten raw	Always	39	8.9
	Occasionally or never	372	91.1
Fertilization pattern	Directly	20	4.6
	After disposed	374	95.4
Raised animals	Yes	338	77.2
	No	100	22.8
Wash hands before meals	Always	5	1.2
	Occasionally or never	427	98.8
Source of drinking water	Spring	415	94.7
	Other	23	5.3
Toilets	None	188	42.9
	Yes	250	57.1
Village No.	1	114	26.0
	2	115	26.3
	3	88	20.1
	4	121	27.6

RMB, Renminbi, the current rate of exchange be 9.66 yuan to 1 pound, i.e., ¥9.66 yuan (RMB) = £1 lb (GBP)

**Table 2:** Infection rates of HBV, *Ascaris lumbricoides* and *Trichuris trichiura*

	No. of participants	No. of infected	Infection rate (%)
HBV	438	40	9.1
*A. lumbricoides*	438	59	13.5
*T. trichiura*	438	134	30.6
Co-infected with HBV and *A. lumbricoides*	438	10	2.3
Co-infected with HBV and *T. trichiura*	438	12	2.7

Table 3 shows the risk factors for HBV. Logistic regression analysis showed that age (21–30 years versus > 50 years: OR = 6.66, 95 % CI = 2.15–20.68), drug abuse (OR = 6.96, 95 % CI = 1.11–43.90), *A. lumbricoides* infection (OR = 3.60, 95 % CI = 1.48–8.75), fertilization with faeces after disposal (OR = 0.15, 95 % CI = 0.04–0.47) and work on farm (OR = 4.59, 95 % CI = 1.44–14.63) were factors significantly associated with HBV infection.

**Table 3:** Results of the univariate and multivariate analyses for risk factors associated with HBV infection of the study population

Variables/ states		No. of participants	No. of infections(Rate in %)	cOR (95 % CI)	aOR (95 % CI)
Age (years)	6–10	54	5 (9.3)	1.53 (0.44, 5.27)	1.50 (0.32, 6.92)
	11–20	48	2 (4.2)	0.65 (0.13, 3.36)	1.01 (0.18, 5.69)
	21–30	66	16 (24.2)	4.80 (1.77, 13.05)*	6.66 (2.15, 20.68)**
	31–40	86	8 (9.3)	1.54 (0.51, 4.63)	1.37 (0.41, 4.63)
	41–50	88	3 (3.4)	0.53 (0.13, 2.18)	0.55 (0.12, 2.52)
	>50	96	6 (6.3)	1.00	1.00
Gender	Male	172	14 (8.1)	0.82 (0.41, 1.62)	
	Female	266	26 (9.8)	1.00	
Occupation	Worker	4	0 (0)	0	
	Peasant	370	37 (10.0)	2.26 (0.68, 7.56)	
	Else	64	3 (4.7)	1.00	
Education	Illiterate	286	25 (8.7)	0.88 (0.45, 1.71)	
	Elementary school and above	152	15 (9.9)	1.00	
Marital status	Unmarried	100	8 (8.0)	1.00	
	First married	293	28 (9.6)	1.22 (0.54, 2.76)	
	Divorced and other	45	4 (8.9)	1.12 (0.32, 3.94)	
Home income (¥ yuan, RMB)	<1,000	12	0 (0)	0	
	1,000–2,999	71	3 (4.2)	0.36 (0.10, 1.29)	
	3,000–4,999	142	15 (10.6)	0.97 (0.45, 2.07)	
	5,000–9,999	75	7 (9.3)	(0.33, 2.17)	
	≥10,000	138	15 (10.9)	1.00	
Drug abuse	Yes	9	3 (33.3)	5.30 (1.27, 22.05)*	6.96 (1.11, 43.90)**
	No	429	37 (8.6)	1.00	1.00
Work outside	Yes	134	17	1.78 (0.92, 3.45)	
	No	304	23	1.00	
Work on farm	Yes	265	32 (12.1)	2.18 (0.97, 5.07)	4.59 (1.44, 14.63)**
	No	113	5 (4.4)	1.00	1.00
Clean food when eaten raw	Always	39	0 (0)	1.00	
	Occasionally	244	28 (11.5)	0	
	Never	128	9 (7.0)	1.71 (0.78, 3.75)	
Fertilization pattern	Directly	20	6 (30.0)	1.00	1.00
	After disposal	374	31 (8.3)	0.21 (0.08, 0.59)*	0.14(0.04,0.47)**
Raising Animals	Yes	338	34 (10.1)	1.75 (0.71, 4.30)	
	No	100	6 (6.0)	1.00	
Toilets	None	188	19 (10.1)	0.852 (0.43, 1.57)	
	Yes	250	21 (8.4)	1.00	
Village No.	1	114	16 (14.0)	1.81 (0.79, 4.18)	
	2	115	5 (4.4)	0.51 (0.17, 1.52)	
	3	88	9 (10.2)	1.27 (0.49, 3.26)	
	4	121	10 (8.3)	1.00	
*A. lumbricoides*	Negative	379	30 (7.9)	1.00	1.00
	Positive	59	10 (17.0)	2.37 (1.09, 5.16)*	3.60 (1.48, 8.75)**
*T. trichiura*	Negative	304	28 (9.2)	1.00	
	Positive	134	12 (9.0)	0.97 (0.48, 1.97)	

*CI* confidence interval, *OR* odds ratio, *cOR* crude odds ratio, *aOR* adjusted odds ratio, *HBV* hepatitis B virus

*RMB* Renminbi, the current rate of exchange be 9.66 yuan to 1 pound, i.e., ¥ 9.66 yuan (RMB) = £1 lb (GBP)

*, *P* ≤ 0.05 in univariate analyses; **, *P* ≤ 0.05 in and multivariate analyses

Table 4 shows the risk factors for *A. lumbricoides*. Multivariate logistic regression analysis showed that village (village 1 versus village 4: OR = 3.14, 95 % CI = 1.35–7.27), HBV infection (OR = 2.55, 95 % CI = 1.07–6.16) and infection with *T. trichiura* (OR = 3.11, 95 % CI = 1.63–5.93) were positively associated with *A. lumbricoides* infection. In addition, having toilets at home was negatively related to *A. lumbricoides* infection (OR = 0.52, 95 % CI = 0.27–0.98).

**Table 4:** Results of the univariate and multivariate analyses for risk factors associated with *Ascaris lumbricoides* and *Trichuris trichiura* infections of the study population

			*A. lumbricoides*			*T. trichiura*		
Variables/ states		No. of participants	No. of infections (Rate in %)	cOR (95 % CI)	aOR (95 % CI)	No. of infections (Rate in %)	cOR (95 % CI)	aOR (95 % CI)
Age (years)	6–10	54	7 (13.0)	1.04 (0.38, 2.83)		22 (40.7)	1.95 (0.96, 3.97)*	1.69 (0.73, 3.93)
	11–20	48	7 (14.6)	1.20 (0.44, 3.26)		21 (43.8)	2.21 (1.06, 4.58)*	3.72 (1.59, 8.67)**
	21–30	66	7 (10.6)	0.83 (0.31, 2.34)		17 (25.8)	0.99 (0.48, 2.02)	1.24 (0.54, 2.82)
	31–40	86	13 (15.1)	1.25 (0.54, 2.90)		28 (32.6)	1.37 (0.72, 2.60)	1.61 (0.77, 3.36)
	41–50	88	13 (14.8)	1.21 (0.52, 2.82)		21 (23.9)	0.89 (0.46, 1.74)	1.01 (0.47, 2.18)
	>50	96	12 (12.5)	1.00		25 (26.0)	1.00	1.00
Gender	Male	172	23 (13.4)	0.99 (0.56, 1.73)		55 (32.0)	1.13 (0.74, 1.68)	
	Female	266	36 (13.5)	1.00		79 (29.7)	1.00	
Wash hands before meals	Always	5	0 (0)	0.00		1 (20.0)	0.60 (0.06, 5.05)	
	Occasionally or never	427	59 (13.8)	1.00		132 (30.9)	1.00	
Source of drinking water	Spring	415	57 (13.7)	1.21 (0.35, 4.15)		131 (31.6)	1.50 (0.59, 3.83)	
	Other	23	2 (8.7)	1.00		3 (13.0)	1.00	
Work on farm	Yes	305	40 (13.1)	0.91 (0.50, 1.63)		90 (29.5)	0.85 (0.50, 1.31)	
	No	133	19 (14.3)	1.00		44 (33.1)	1.00	
Clean food when eaten raw	Always	39	8 (20.5)	2.10 (0.81, 5.46)		16 (41.0)	1.53 (0.73, 3.21)	
	Occasionally	244	33 (13.5)	1.27 (0.60, 2.48)		72 (29.5)	0.92 (0.58, 1.46)	
	Never	128	14 (10.9)	1.00		40 (31.3)	1.00	
Fertilization pattern	Directly	20	5 (25.0)	1.00		6 (30.0)	1.00	
	After disposal	374	49 (13.1)	0.45 (0.16, 1.30)		112 (30.0)	1.00 (0.37, 2.66)	
Animal raise	Yes	338	48 (14.2)	1.34 (0.67, 2.69)		106 (31.4)	1.18 (0.72, 1.92)	
	No	100	11 (11.0)	1.00		28 (28.0)	1.00	
Toilets	None	188	35 (18.6)	1.00	1.00	69 (36.7)	1.00	1.00
	Yes	250	24 (9.6)	0.46 (0.27, 0.81)*	0.51 (0.27, 0.98)**	65 (26.0)	0.61 (0.40, 0.91)*	0.48 (0.28, 0.80)**
Village No.	1	114	25 (21.9)	3.12 (1.42, 6.83)*	3.14 (1.35, 7.27)**	29 (25.4)	1.63 (0.86, 3.06)	1.34 (0.64, 2.79)
	2	115	19 (16.3)	2.20 (0.97, 4.95)*	1.61 (0.67, 3.89)	48 (41.7)	3.41 (1.87, 6.21)*	3.73 (1.92, 7.26)**
	3	88	5 (5.7)	0.67 (0.22, 2.03)	0.71 (0.21,2.36)	36 (40.9)	3.30 (1.75, 6.22)*	4.53 (2.12, 9.68)**
	4	121	10 (8.3)	1.00	1.00	21 (17.4)	1.00	1.00
HBV	Negative	398	49 (12.3)	1.00	1.00	122 (30.7)	1.00	
	Positive	40	10 (25.0)	2.37 (1.89, 5.16)*	2.55 (1.07, 6.06)**	12 (30.0)	0.97 (0.48, 1.97)	
*A. lumbricoides*	Negative	379				103 (27.2)	1.00	1.00
	Positive	59				31 (52.5)	2.97 (1.70, 5.19)*	3.09 (1.62, 5.92)**
*T. trichiura*	Negative	304	28 (9.2)	1.00	1.00			
	Positive	134	31 (23.1)	2.97 (1.70, 5.19)*	3.11 (1.63, 5.93)**			

*CI* confidence interval, *OR* odds ratio, *cOR* crude odds ratio, *aOR* adjusted odds ratio, *HBV* hepatitis B virus

*, *P* ≤ 0.05 in univariate analyses; **, *P* ≤ 0.05 in and multivariate analyses

Table 4 shows the risk factors for *T. trichiura*. After adjustment in multivariate logistic regression analysis, village (village 2 versus village 4: OR = 3.73, 95 % CI = 1.97–7.26); village 3 versus 4: OR = 4.53, 95 % CI = 2.12–9.68), *A. lumbricoides* infection (OR = 3.09, 95 % CI = 1.62–5.92) and age (11–20 years versus >50 years: OR = 3.72, 95 % CI = 1.59–8.67) were significantly associated with *T. trichiura* infection. Having toilets at home was negatively associated with *T. trichiura* infection (OR = 0.48, 95 % CI = 0.28–0.80).

Stratification analysis by village showed that only gender (female versus male, OR = 4.65, 95 % CI = 1.25–17.27) and toilets (OR = 0.32, 95 % CI = 0.10–0.98) were related with *A. lumbricoides* in village 2, and no risk factors associated with *A. lumbricoides* were found in other villages. However, different risk factors for *T. trichiura* were detected in different villages. Related risk factors of *T. trichiura* were not found in village 1 while toilets (having toilets was negatively related with *T. trichiura*, OR = 0.25, 95 % CI = 0.11–0.54), gender (female was negatively associated with *T. trichiura* versus male, OR = 0.52, 95 % CI = 0.17–0.98) and age (age ranges of 6–10 years and 11–20 years were positively associated with *T. trichiura* compared with age above 50 years, OR = 14.25, 95 % CI = 1.42–143.19 and OR = 14.25, 95 % CI = 2.07–98.14) were found associated with *T. trichiura* in village 2, village 3 and village 4 separately.

### Discussion

This study reported the prevalence of HBV, *A. lumbricoides* and *T. trichiura* infections*.* The prevalence of HBV and *A. lumbricoides* infections were comparable to the national level while *T. trichiura* was higher than the national level [10, 13, 14]. Poor hygiene may be a major reason. Most people in this area drank unboiled water (91.8 %, 402/438) and ate raw food without washing (89.1 %, 366/411). *Trichuris trichiura* is mainly transmitted by contaminated water and food [15]. Furthermore, the altitude (1,800–2,500 m) and temperature (10–22 °C) were shown to be suitable for *T. trichiura* [12, 16].

Drug abuse and age were positively associated with HBV infection, which was consistent with the results previously reported [17, 18]. Having toilets at home and villages were independently associated with *A. lumbricoides* infection, and toilets, villages and age were independently associated with *T. trichiura*. Having toilets was the common protective factor for *A. lumbricoides* and *T. trichiura*. Previous studies in China showed that having toilets at home was associated with a lower risk of parasite infection [19]. Improvement of sanitation condition is essential to reduce the risk of *A. lumbricoides* and *T. trichiura* infections.

We noted that people had different risks of *A. lumbricoides* and *T. trichiura* infections in different villages. We therefore carried out a stratified analysis for *A. lumbricoides* and *T. trichiura* infections according to the villages and adjusted by all of their potential risk factors. We found different risk factors for helminthic infections in different villages. This might be the reason why people had different prevalences of *A. lumbricoides* and *T. trichiura* in different villages.

We found no significant differences between gender, occupation, race, education or household income and HBV, *A. lumbricoides* and *T. trichiura*. First of all, restricted to the study field, 96.8 % of the study population were Yi people and therefore may restrict the statistical power to distinguish the meaning of race in this relationship. Besides, we selected people with the same criteria like aged 6 years or over, living in the area for more than 6 months every year. This means even though they had different occupations, education levels and home incomes, they had a similar way of life. So people included in this study might have similar characteristics in living, eating and drinking habits. They were exposed to the same culture and environmental surroundings, and were limited in the same resident scope. So the above might provide the explanation.

Some studies have reported that co-infection with HBV could accelerate the progression of schistosomiasis and make its treatment more complex and difficult, and *vice versa* [20, 21]. It might be expected that other helminths such as *A. lumbricoides* and *T. trichiura* might produce similar immunoreaction in humans. However, unlike *S. japonicum,* which can cause severe disease, *A. lumbricoides* or *T. trichiura* that inhabit the intestinal tract of humans are non-pathogenic parasites or only result in mild illnesses. Intestinal helminths most commonly cause diseases in immunocompromised individuals, such as people with HBV, human immunodeficiency virus (HIV) infections and other severe diseases [22].

We found association between infections with HBV and *A. lumbricoides*. HBV might be a risk factor for *A. lumbricoides* infection, or infection with *A. lumbricoides* could increase the risk of HBV infection. HBV may cause an imbalance between Th1 and Th2 cells, and a study found that Th1 cells were suppressed while Th2 cells were enhanced [23]. Shift from Th1 to Th2 cells was also seen in parasite infections [24]. A balance of these two types of cells was important for the body’s resistance to various pathogens. An interesting question to consider is whether immune responses for helminthic infection are facilitated by the replication of HBV DNA and if HBV infection contributes to a deterioration in helminthic infection. It is worthwhile further investigating how host immune response changes when co-infected with HBV and helminths.

We found that HBV infection was associated with fertilization pattern and farm work activities. Fertilization with faeces after disposal (i.e. faeces processed with fermentation, drying and other hazard-free treatments) was related to a lower prevalence of HBV infection compared with fertilization directly (i.e. no treatment of faeces before fertilisation) (8.3 % versus 30.0 %, OR = 0.21, 95 % CI = 0.08–0.59). HBV DNA exists in faeces of people with HBV infection, i.e. faeces of these people were infectious for HBV. This result is consistent with studies conducted by Guo et al*.* and Zhou et al*.* [25, 26]. The latter found that HBV DNA load was up to 2.245 × 108 copies/ml in faeces, indicating that faeces could be the medium of HBV transmission. Thus it is understandable that working on a farm was related with HBV infection. People in this area were almost all barefoot while conducting farm work.

To prevent and control infections of *A. lumbricoides* and *T. trichiura*, the most effective measurement was to establish toilets or any other form of sanitation facilities drawn from our investigation. Strengthening health education for school children and taking preventive anthelmintic treatment are other effective ways. As to HBV infection, drug prohibition was the essential measurement and we found the key was implementing non-hazardous treatment of faeces and wearing shoes when working on farms in this area. They were also protective for people against helminthic infections.

There were several limitations for the study. The excluded participants were generally younger than those included in this study because a larger number of young people left for cities to find jobs. Potential biases might result in an underestimation of the prevalence of HBV infection as young adults were at higher risk for HBV infection [27]. Some information was lacking including the length of time spent working on a farm. We could not establish a dose-response relationship between this and HBV infection. Because of the nature of cross-sectional design, we were not able to give the affirmative conclusion for the relationship between HBV infection and *A. lumbricoides*. The sample size was not enough for performing more detailed stratified statistical analysis by villages. In view of these limitations, the findings should be interpreted with caution. We did not find a significant association between HBV infection and *T. trichiura*, which should have a similar to *A. lumbricoides* transmission route and pathogenicity.

## Conclusions

In conclusion, drug abuse, age of 21–30 years and infections with *A. lumbricoides* were independently associated with HBV infection. Having toilets was independently associated with a reduced risk of *A. lumbricoides* and *T. trichiura* infections. Working on a farm and fertilization with faeces without disposal were positively associated with HBV infection. Improving hygiene conditions and habits, particularly having toilets at home, are essential for reducing the risks of *A. lumbricoides* and *T. trichiura* infections.

## Abbreviations

CI: confidence interval
DALYs: disability-adjusted life years
DNA: deoxyribonucleic acid
HBV: Hepatitis B Virus
HIV: human immunodeficiency virus
OR: odds ratio
STH: soil-transmitted helminth
